# Radioembolization of Hepatic Lesions from a Radiobiology and Dosimetric Perspective

**DOI:** 10.3389/fonc.2014.00210

**Published:** 2014-08-19

**Authors:** Marta Cremonesi, Carlo Chiesa, Lidia Strigari, Mahila Ferrari, Francesca Botta, Francesco Guerriero, Concetta De Cicco, Guido Bonomo, Franco Orsi, Lisa Bodei, Amalia Di Dia, Chiara Maria Grana, Roberto Orecchia

**Affiliations:** ^1^Istituto Europeo di Oncologia, Milan, Italy; ^2^Istituto Nazionale dei Tumori, Milan, Italy; ^3^Istituto Nazionale dei Tumori Regina Elena, Rome, Italy; ^4^Istituti Ospitalieri di Cremona, Cremona, Italy; ^5^Istituto Candiolo – IRCCS, Candiolo, Italy

**Keywords:** radioembolization, liver tumors, ^90^Y-microspheres, dosimetry, radiobiology

## Abstract

Radioembolization (RE) of liver cancer with ^90^Y-microspheres has been applied in the last two decades with notable responses and acceptable toxicity. Two types of microspheres are available, glass and resin, the main difference being the activity/sphere. Generally, administered activities are established by empirical methods and differ for the two types. Treatment planning based on dosimetry is a prerogative of few centers, but has notably gained interest, with evidence of predictive power of dosimetry on toxicity, lesion response, and overall survival (OS). Radiobiological correlations between absorbed doses and toxicity to organs at risk, and tumor response, have been obtained in many clinical studies. Dosimetry methods have evolved from the macroscopic approach at the organ level to voxel analysis, providing absorbed dose spatial distributions and dose–volume histograms (DVH). The well-known effects of the external beam radiation therapy (EBRT), such as the volume effect, underlying disease influence, cumulative damage in parallel organs, and different tolerability of re-treatment, have been observed also in RE, identifying in EBRT a foremost reference to compare with. The radiobiological models – normal tissue complication probability and tumor control probability – and/or the style (DVH concepts) used in EBRT are introduced in RE. Moreover, attention has been paid to the intrinsic different activity distribution of resin and glass spheres at the microscopic scale, with dosimetric and radiobiological consequences. Dedicated studies and mathematical models have developed this issue and explain some clinical evidences, e.g., the shift of dose to higher toxicity thresholds using glass as compared to resin spheres. This paper offers a comprehensive review of the literature incident to dosimetry and radiobiological issues in RE, with the aim to summarize the results and to identify the most useful methods and information that should accompany future studies.

## Introduction

In the past two decades, radioembolization (RE) with ^90^Y-microspheres has emerged as a safe and efficacious treatment modality for unresectable primary and secondary liver malignancies. The rationale is based on the fact that both the primary and the secondary tumor (*T*) lesions in the liver receive their blood supply primarily from the hepatic artery, whereas non-tumoral liver (NL) is almost excluded from the hepatic artery and fed essentially via the portal vein. Spheres are injected in the hepatic artery and become trapped within the *T* microvasculature, so that they selectively deliver radiation to the *T* whilst sparing normal tissue. In NL, microspheres remain confined to the portal tracts (1). This locoregional technique represents a good option to irradiate liver *T* as compared to external beam radiation therapy (EBRT), this latter being limited by the high radiosensitivity of the liver parenchyma. A further advantage of RE is that it can be combined with other therapy modalities that are under study in several phase III multicentre trials (2). Two types of microspheres are commercially available, the glass spheres [Therasphere^®^ BTG, Ontario, Canada (3)] and the resin spheres (SIR-Spheres^®^, SIRTex Medical Limited Sydney, Australia, http://www.sirtex.com) (4). The activity to be administered is chosen using empirical or raw dosimetric methods, depending on the types of spheres, while fully dosimetric treatment planning is very rarely applied. Overall, significant response rates have been observed in patients with unresectable primary or secondary hepatic malignancies, with a limited number of side effects (5–8). However, no study was specifically accomplished to enlighten the power of a fully dosimetric treatment planning: increased efficacy and reduced toxicity. In nuclear medicine treatment, the importance of dosimetry to comprehend the radiobiological effects is growing and documented by the increased number of papers addressing dosimetry in the recent literature (9–13). In RE, the essential dosimetry methods based on compartmental models have been more traditionally applied and have proved correlations between absorbed doses and effects. Even if still in few centers, dosimetry planning guides RE treatment (13–15). Specific experimental studies and mathematical modeling at the microscopic level have provided enlightening information for the understanding of macroscopic observations, such as the RE-induced liver disease (REILD) and the different behavior of glass versus resin spheres, with the apparent higher tolerability of RE with glass spheres and higher absorbed doses associated to *T* response (12–14, 16). More recently, 3D voxel dosimetry methods have been applied to RE, providing dose distribution maps and dose–volume histograms (DVH). This allowed to apply radiobiological models to predict the probability of toxicity, normal tissue complication probability (NTCP), and *T* response, tumor control probability (TCP). In regard to the clinical context, outcomes such as the volume effect, the influence of the functional reserve and/or concomitant therapies, with higher tolerability of re-treatment, reflect a further similarity with EBRT, which can be identified as a foremost reference to compare with, although differences with EBRT exist and must be considered. The aim of this study is to comprehensively review the literature addressing these issues from a radiobiological perspective.

## Two Types of ^90^Y-Microspheres

^90^Y is a pure β-emitter radionuclide with maximum and average β-energies of 2.28 MeV and 934 keV, respectively, corresponding to a maximum and average path lengths in soft tissue of 11 and 4.1 mm, respectively. It has a half-life of 64.2 h and it decays into the stable element ^90^Zr. There is also a minor branch of the decay to a O + first excited state of ^89^Zr at 1.78 MeV, which is followed by a β + β-emission. Although the branching ratio is very low (32 × 10^−6^), it is most important as it allows ^90^Y-microspheres PET imaging after RE (17, 18). The release of the absorbed dose occurs in the surrounding tissue according to the ^90^Y-range.

The main characteristics of glass and resin spheres are summarized in Table 1. Microspheres are biocompatible but not biodegradable or metabolized. The most relevant difference between the two types of spheres is the activity per sphere, which is much higher in the glass spheres (~2500 Bq) than in resin ones (~50 Bq) (see also section Activity Distribution at the Microscopic Level). A further difference between resin and glass is the shelf-life, being 1 day for resin spheres, and 12 days for glass spheres. This implies that the same number of resin spheres/GBq is always injected, while the number of glass spheres/GBq increases according to ^90^Y physical decay (time interval left between preparation and administration). Overall, for a same activity, the ratio between the number of resin and glass spheres can vary from 50 (2500 Bq/50 Bq) to 2.2 [2500 Bq/(22.6 × 50) Bq]. This point has interesting dosimetric consequences that will be described later on (section Activity Distribution at the Microscopic Level).

**Table 1:** **Characteristics of ^90^Y-microspheres (6, 20, 24)**.

Commercial name	SIR-Spheres^®^	TheraSphere^®^
Manufacturer	Sirtex Medical, Lane Cove Australia	Therasphere BTG, Ontario, Canada
Material	Resin	Glass
^90^Y sphere production	Bound to resin, attached to sphere surface	Embedded in a glass matrix
Particle size (μm)	32.5 ± 2.5 (range: 20–60)	25 ± 5 (range: 20–30)
Activity per sphere (Bq)	50 (range: 40–80)	2500 at the reference time
Number of spheres per GBq (million)	20 (mean)	0.4 at the reference time
Shelf-life	1 day	12 days
Specific gravity	Low (1.6 g/cc)	High (3.6 g/cc)
Embolic effect	Moderate	Mild
Activity available (GBq)	3	From 3 to 20, with step 0.5
Number of spheres in 3GBq	40–80 million	1.2 million at the time of calibration
Approved for	USA: HCC; Outside USA (especially Europe and Australia): unresectable liver tumors (HCC and metastases)	USA: colorectal carcinoma Outside USA (especially Europe and Australia): HCC and metastases
Handling for dispensing	Required	Not possible
Splitting one vial for two or more administrations	Possible	Not possible
Necessity of contrast medium guidance during administration	Yes	No

Flow stasis, making the injection of the whole planned activity impossible, has been observed during resin microspheres administration (especially in super-selective treatments), while it has never been reported with glass microspheres (19, 20). This is probably due to the very high number of particles, and suggests a possible role of embolization in the case of resin spheres (see also section Activity Distribution at the Microscopic Level).

## Administered Activity

The amount of activity to be administered should ideally be established accounting for the major factors that may influence therapy outcome. Among these, there are the NL involvement, the tumor uptake, the possible side effects to healthy tissues, the baseline patients’ condition, and the absorbed dose. More rarely, the activity to be injected is based on dosimetric evaluations (13–15, 21, 22). Table 2 summarizes the methods proposed, equations, constraints, and essential pros and cons for the two types of microspheres.

**Table 2:** **Methods to determine the activity to be injected according to device user manual**.

Sphere type	Model and references	*A* (GBq)		Notes
		General cases	Special constraints	
Resin	(i) Empirical (4, 24)	In case of LS < 10% 2.0 if tumor involvement ≤ 25% 2.5 if tumor involvement 25–50% 3.0 if tumor involvement > 25–50	Activities lowered by 20% if LS = 10–15%; 40% in case LS = 15–20%; treatment avoided if LS > 20%	Empirical; not personalized for different liver size nor for tumor and liver perfusion; based on whole liver infusion
	(ii) BSA (4, 24)	In case of LS < 10% $\text{A}=\left(\text{BSA}-0.2\right)+\frac{{\text{M}}_{\text{T}}}{{\text{M}}_{\text{T}}+{\text{M}}_{\text{NL}}}$	Activities lowered by 20% if LS 10–15%; 40% if LS 15–20%; No treatment if LS > 20%	Empirical; the most commonly applied; heritage from chemotherapy; not actual personalization; individual tumor and parenchyma perfusion not accounted; more conservative than empirical method; based on whole liver infusion; prescription empirically reduced for safer approach
	(iii) Partition or multi-compartmental method (4, 15, 26, 27)	$\text{A}={\text{D}}_{\text{NL}}\times \frac{\text{T/NL}\cdot {\text{M}}_{\text{T}}+{\text{M}}_{\text{NL}}}{50\cdot (1-\text{LS})}$ with $\text{T/NL}=\frac{{\text{A}}_{\text{T}}}{{\text{M}}_{\text{T}}}∕\frac{{\text{A}}_{\text{NL}}}{{\text{M}}_{\text{NL}}}$	Activities lowered by 20% if LS 10–15%; 40%ifLS 15–20%; No treatment if LS > 20%; *D*_NL_ = 80 Gy; 70 Gy if cirrhosis *D*_lungs_ < 25 Gy, preferably 20 Gy	Personalized, accounts for tumor avidity and liver involvement; based on whole liver infusion, originally designed for single or discrete nodules, appropriate modification by makes it applicable to multiple lesions
Glass	(iv) Monocom-partmental MIRD (partitional) (3, 28)	$\text{A}=\frac{{\text{D}}_{\text{M}}\times \text{M}}{50\left(1-\text{LS}\right)}$	Constraint *D*_M_ to the whole tissue treated, typically 80–150 Gy, and constraint of *D*_lungs_ (typically 20–30 Gy) <10% shunt	Not really personalized, it does not discriminate for different tumor avidity and involvement; applicable to whole liver, lobar, and segmental infusion

**A*, activity (GBq); LS, lung shunt fraction; BSA, body surface area; *A*, activity to be administered (GBq); *M*_T_, tumor mass (kg); NL, non-tumoral liver; *T*, tumor; *M*_NL_, NL mass (kg); *A*_T_, *T* uptake (GBq); *A*_NL_, NL uptake (GBq); *T*/NL, *T* over NL uptake ratio; *D*_NL_, absorbed dose limit prescribed for NL (Gy); *D*_lungs_, absorbed dose limit prescribed for the lungs (Gy); 50, conversion factor (Gy × kg/GBq) for ^90^Y for a standard patient; *M*, whole mass (*M*_NL_ + *M*_T_) (kg); *D*_M_, absorbed dose limit prescribed for *M**.

For resin microspheres, three methods (i, ii, and iii) have been suggested by the manufacturer (4) to decide the ^90^Y activity. In common, they have the indication of reducing the prescribed activity depending on the lung shunt (LS) fraction, in order to lower the radiation risks to the lungs.

(i) *The empirical method* recommends three different activities based on *T* involvement (Table 2). It is not an individualized approach because it does not account for the volume of the NL involved in the irradiation, and thus its tolerability. It is worth noting that with the highest activity, the absorbed doses to the lungs and to NL are within ~18 and ~83 Gy, as derived using the OLINDA/EXM software for a standard patient (23). Note that among 28 patients dead at least in part because of liver toxicity over 680 treated with resin spheres, 21 from a single center were administered according to this method (24).
(ii) *The body surface area (BSA)* method, still empirical, takes into account the patient’s BSA, the *T* mass (*M*_T_) and the NL mass (*M*_NL_), assuming a correlation between BSA and *T*. However, there is experimental evidence that the BSA does not correlate with *M*_L_ or with *T* involvement (25). Even if it includes some individual parameters, it should not be misconstrued in terms of tailored evaluation, as it neglects the individual *T*/NL avidity ratio, which is patient specific, even lesion specific (22). Activities remain usually within 2.5 GBq (25). This is the method most commonly used for RE with resin spheres (24).
(iii) *The multi-compartmental MIRD macrodosimetry* method (also known as partition model) (26, 27) calculates the activity to be administered using the MIRD equations, once an absorbed dose limit is prescribed to NL, but no liver toxicity nor efficacy threshold accompanied this methodology. The lung safety is also considered, with a specific absorbed dose constraint. The equation takes into account the *T*/NL uptake ratio, the liver involvement (*M*_T_ and *M*_NL_) and the possible LS.

One single method is proposed for glass spheres by the manufacturer:

(iv) The *mono-compartmental MIRD macrodosimetry* model relies on a simplified dosimetry equation of the multi-compartmental model (3, 7, 28) in which an absorbed dose to the whole liver or lobe (*D*_M_) – typically ranging from 80 to 150 Gy – is empirically prescribed as averaged on the whole treated liver or lobe, including the *T*, of mass *M* = *M*_T_ + *M*_NL_. So, a uniform activity distribution is assumed and absorbed doses to *T* and NL are not separately calculated. No distinction is made for different tumor involvement and avidity.

The methods (i), (ii), and (iv) are appealing for their simplicity but clearly suboptimal. This has led to possible under-treatments, compromising efficacy, or over-treatments, inducing unwanted effects. The multi-compartmental dosimetric model (iii) provides a more scientifically sound basis for the activity determination, although it demands a confident identification of absorbed doses ensuring liver tolerability. Moreover, it relies on the possibility to simulate therapy.

## Simulation of the Treatment

The therapy session is generally simulated with intra-arterial administration of ^99m^Tc-MacroAggregate Albumin (^99m^Tc-MAA) that should mimic the vascular distribution pattern of ^90^Y-microspheres. The simulation is mandatory to evaluate the possible LS fraction and to exclude gastrointestinal (GI) shunt. ^99m^Tc-MAA is also used to analyze, at least visually (29), the *T*/NL uptake ratio and to evaluate dosimetry in the frame of individualized treatment planning. This is based on the hypothesis that ^99m^Tc-MAA and ^90^Y-microspheres have identical intra-hepatic distribution, due to comparable size (10–100 μm diameter) and density (12, 26). However, the adequacy of this hypothesis is under debate, since the number/dimension of injected resin, glass, and MAA particles differ. The number of injected resin spheres greatly differ, being ~300 times higher than the number of ^99m^Tc-MAA particles and ~50 times higher than the number of glass spheres (12). Thus, a possible higher embolic effect of resin spheres is expected as compared to glass spheres, versus no significant embolic effect of MAA particles on the hepatic arterial circulation. Other differences may rise from different micro-catheter tip placement and regional blood flow changes between simulation and RE. These points have created concern about the predictive value of ^99m^Tc-MAA scintigraphy for NL and *T* dosimetry. Results from the literature are controversial. Wondergem et al. (30) found discrepancies between ^99m^Tc-MAA images simulating RE and ^90^Y-Bremsstrahulg SPECT images – post-RE – in patients with a mismatch in catheter tip positioning during administrations. This study highlights the importance of this maneuver when using MAA images as predictive of microsphere distribution. Knesaurek et al. (31) found poor correlation in some of the patients analyzed. The same group confirmed the critical role of catheter repositioning in combination with proximity to an arterial bifurcation (32). In a study object of discussion, Ulrich et al. claimed a low predictivity of MAA based on visual examination of MAA and ^90^Y-Bremsstrahulg SPECT images (29, 33). On the other hand, despite undeniable differences, the significance of ^99m^Tc-MAA in the treatment planning of resin spheres has been shown (14, 33, 34). Strigari et al. (16) stated that ^99m^Tc-MAA SPECT images of the abdomen were sufficiently predictive of the ^90^Y-SIR sphere distribution in more than 80% of patients. Flamen et al. (35) reported that MAA and ^90^Y imaging did correspond in all cases.

For glass spheres, the problem is less evident, thanks to the lower number of injected particles. The capability of MAA based lesion dosimetry to predict response and even overall survival (OS) has been shown (14), meaning that in the majority of patients the simulation is worthwhile (33). Chiesa et al. (12) compared ^99m^Tc-MAA and ^90^Y-SPECT in 35 patients treated with glass spheres. In 71% of cases, distributions were similar. In 8/35 patients, differences were marked, and uncertain in 6%. Discrepancies were attributed mostly to intentional changes in catheter positioning, while in 2 patients (6%) to the different specific weight of glass microspheres with respect to MAA. Kao et al. (36) retrospectively analyzed ^99m^Tc-MAA-SPECT and ^90^Y-PET images, and compared the mean *T* absorbed doses. Excellent correlation was found in selected patients, with a relative error ranging from −1.2% to +13.2%. Preliminary results from direct DVH comparison between ^99m^Tc-MAA-SPECT and ^90^Y-PET have been presented to assess the role of treatment planning based on simulation images (37, 38). Several authors showed a good correlation between the *T* absorbed dose based on the partition model and the response to ^90^Y-microspheres in metastatic disease, as well as in HCC (12, 14, 22, 35, 39–41). In particular, Garin et al. (14) have shown that dosimetry based on ^99m^Tc-MAA-SPECT not only predicts *T* response but also OS in patients with HCC. ^99m^Tc-MAA scintigraphy is a relevant source of information for patient recruitment and RE treatment feasibility. Provided that both the tracer and microspheres are injected under the same condition, ^99m^Tc-MAA SPECT-CT gives an accurate description of the microspheres distribution in *T*, as well as in normal tissues, predicting the shunt to extra-hepatic lungs and the GI tract (36, 42, 43).

## Verification of the Treatment

The evaluation of the actual biodistribution in RE is of utmost importance in order to verify the ^99m^Tc-MAA prediction. A first option is to acquire Bremsstrahlung-SPECT images (12, 16, 43, 44), although difficulties raise from the poor-quality images that could lead to inaccurate quantification of microsphere biodistribution. Some authors have shown the possibility to obtain impressive imaging improvements by applying appropriate corrections for scatter, attenuation, and response of the system (45, 46) or a special collimator (47), but the suggested methods are applied only in very few research centers since they require very careful calibration and experience. Alternatively, Lhommel et al. (17), followed by other authors (18, 48–50), have shown that PET/CT scanners are able to provide good-quality ^90^Y images when radioactivity is highly concentrated, as in RE, and that accurate patient dosimetry is attainable. A further step is represented by the 3D analysis at the voxel level. Several authors have elaborated pre-treatment ^99m^Tc-MAA-SPECT and/or post-treatment ^90^Y-PET images (35, 36, 49–52) identifying the heterogeneity of the activity distribution at the spatial resolution of SPECT or PET. Recent studies have applied the voxel dosimetry to ^90^Y-PET images to obtain dose distribution maps and DVH (36, 37, 49, 50, 53). These methods, which recall those used in EBRT, could improve dose–effect correlations and identify appropriate radiobiological models.

## Activity Distribution at the Microscopic Level

The standard dosimetry at the organ level assumes uniform activity in tissues and considers only marginally patient-specific variations. Based on 3D image analysis, the heterogeneity of the activity distribution can be assessed at the spatial resolution of SPECT or PET, which remains, however, at the macroscopic level (35, 36, 49, 51–54). To assess the heterogeneity at the microscopic level, and more deeply understand the radiobiological mechanisms, some authors analyzed the particle distribution of explanted livers (55, 56). Other studies have elaborated mathematical simulations of the hepatic structures to derive the dosimetry at the microscopic scale. This paragraph focuses on the main issues of these studies.

Fox et al. (55) were the first to introduce the basic argument of the non-uniformity of dose distribution at microscopic level, studying the microsphere positions in an explanted treated liver. They found that dose distribution around a microsphere exhibits an extreme dose gradient of more than 5 orders of magnitude in 2 mm and thus the non-uniformity of dose deposition spares regions of parenchyma, increasing its tolerance with respect to EBRT.

Yorke et al. (57) applied a parallel model introducing a microscopic lobule model to account for non-uniform dose deposition at microscopic level and explain the lack of liver complications with liver absorbed doses up to 150 Gy (58). Dose-rate effects were also introduced and variable values of the enzymatic halftime of sub-lethal damage repair (*T*_rep_ = 0.5, 1, 1.5, 2 h) were considered. Interestingly, TD5 and TD_50_ values increased by more than 50 Gy compared to the uniform dose distribution, confirming the impact of non-uniformity.

Ten years later, Kennedy et al. (56), provided the pathologic hepatic findings in four explanted livers, performing dosimetry on a microscopic scale in a *T* irradiated with glass microspheres. The authors found a preferential and heterogeneous deposition of microspheres at the edge of *T* nodules compared with the center of the tumor or NL, with a ratio from 3:1 to 20:1. The consequence is a selective radiation delivery to the *T*/NL edge. Resin and glass microspheres showed similar distribution in NL, whilst a higher number of resin spheres tended to cluster at the edge of the *T* nodules. This was imputed to different number of resin and glass spheres administered and to higher specific activity of glass spheres. No veno-occlusive disease (VOD) or radiation hepatitis but only slight radiation effect was observed in NL parenchyma far from the tumors (over the range of the β particles). 3D dose calculations showed that both glass and resin microspheres deliver heterogeneous absorbed doses to the tumor (ranging from 100 Gy to more than 3000 Gy). A rapid dose falloff from 300 to 100 Gy within 4 mm was reported. The two cases of colon metastases showed at least 90% necrosis in all tumor nodules. Overall, the findings confirmed that both liver tumor (HCC and metastases) are preferentially vascularized by the hepatic artery and that the vasculature is crucial for the efficacy of RE. Vessel density is heterogeneous within the liver and the *T* (6), as the activity distribution, and therefore less effective than in EBRT.

In the paper by Gulec et al. (1), a 3D hexagonal liver model based on lobular microanatomy was developed, and the microscopic absorbed dose distribution was calculated in various components of the NL structure by Monte Carlo code. Spheres in NL are entrapped within the terminal arterioles/portal tracts at the edges of each lobule, which have diameters comparable to those of the microspheres (30 μm). The model represented this pattern considering a hepatic lattice of lobules and the corresponding vascularity, with the portal tract (hepatic artery, portal vein, and bile duct), and the central vein at the center of each lobule. A uniform linear distribution of the microspheres was reproduced within the hepatic artery (no clustering was simulated), with different scenarios including 50 Bq/sphere (resin) and 2500 Bq/sphere (glass). Evaluations according to the macroscopic compartmental model based on MIRD equations were also performed for comparison purposes. The results revealed that average parenchymal and central vein absorbed doses were similar to the average NL absorbed dose of the compartmental model, while the portal tracts received significantly higher absorbed doses. For a single sphere, the central vein received approximately ~6% of the absorbed dose received by the portal tract, compensated by the cross-fire effect to a fraction of ~50%. The major difference observed between the two types of spheres was that for resin spheres, the absorbed dose to portal tract was more than twofold the absorbed dose to the NL, whilst for glass microspheres, the absorbed dose to portal tract could be slightly lower or more than threefold the liver absorbed dose, with marked non-uniformity in portal tracts for glass spheres. Furthermore, it was shown that a significant cross-fire effect increases the absorbed dose to the hepatocellular parenchyma and the central vein. The authors concluded that there was a consistent relationship between the average liver dose as from MIRD macrodosimetry and the microscopic dosimetry estimates. This study validates the clinical utility of the MIRD methodology in the accurate estimation of the absorbed doses to the central vein and parenchyma, but not to the portal tracts. These results are compatible with REILD, as defined by Sangro et al. (1, 59) (see section Side effects to the liver). A strongly non-uniform absorbed dose distribution in portal tracts for glass microspheres is in agreement with the observed higher tolerance.

Another model assessing microscale dosimetry was applied recently by Walrand et al. (60) to explain the apparent paradox of higher liver tolerance to glass (13) as compared to resin spheres (16). Assuming a random microsphere trapping in the portal tracts combined to the different number of spheres, the authors calculated a non-uniform absorbed dose distribution in order to justify the above discrepancies. The same liver model by Gulec et al. (1) was considered, but symmetric and asymmetric branching probabilities of the microspheres were considered at each vessel bifurcation. Simulations were performed leading to absorbed doses of 120 and 40 Gy to the liver for glass and resin microspheres, respectively. The results showed that for a 60–40% branching probability, the fraction of portal tracts without trapped glass microsphere increased, and the lobular dose distribution of glass spheres became strongly asymmetric, with a maximum value of 50 Gy (as compared to 103 Gy of a symmetric spreading). Most importantly, the dose distribution of the portal triads (the critical tissue in RE) of the glass spheres was similar to that of resin microspheres despite a threefold mean absorbed dose difference to the liver. Therefore, the model justifies that the liver tolerance raises from 40 Gy for resin spheres to 120 Gy for glass spheres.

Another interesting point raised by Walrand et al. (60) regards the embolization effect of the portal tracts by the resin microspheres. Without accounting for embolization, a higher number of lobules had at least a portal triad receiving <40 Gy as compared to the case in which embolization was considered: the effect was to redirect a part of the resin microspheres to portal tracts of lower initial trapping probability. Embolization reduces the non-uniformity of the resin microsphere distribution among portal tracts and increases the number of portal tracts receiving more than 40 Gy, with a potentially more toxic treatment. In the clinical applications, the role of embolization with resin spheres is still to be clarified, as controversial results have been published, with a trend toward improved OS of patients with the stasis phenomenon (61), in contrast to only mild inflammation of animal tissues embolized with non-radioactive resin microspheres (62).

Comparing clinical findings with the two kinds of devices, Chiesa et al. (2, 12) suggested that for a fixed mean absorbed dose, the higher the number of particles/GBq, the higher the biological effect. The study by Walrand et al. microscopically interpreted this phenomenon for normal tissue. The distribution at the microscopic scale varies with the number of particles/GBq, increasing toxicity and efficacy, thus new safe mean absorbed doses should be derived (lower with increasing the number of particles) based on future clinical studies. Lewandowski et al. (63) recently reported a study exploring the possible benefits of a “delayed” (an extended shelf-life) treatment with higher number of glass spheres/Gy. In particular, a time shift providing a double number of glass spheres compared to the reference date was considered, keeping the same standard absorbed dose (123 Gy). The authors hypothesized that increasing the number of glass microspheres, a better tumor distribution would occur without additional adverse events. They concluded that this methodology was safe with promising response rate. However, a better understanding of the potential and risk of this approach needs a more systematic comparison among patients having similar clinical status and pathology. Moreover, tumor and NL averaged absorbed dose should be calculated to better clarify the safety and efficacy. Finally, keeping the same rationale of 120 Gy to the lobe should be regarded with caution, being a sort of absorbed dose-escalation study at the microscopic level (especially to the portal tracts), because a higher NL absorbed dose could overcome the threshold for side effects.

## Radiobiological Modeling

The linear quadratic model (LQM) has been used to describe the radiobiological effects in several radionuclide therapies and details can be found in reference (11). More relevant definitions also used in EBRT are introduced. The biological effective dose (BED) is widely used to assess the effect of absorbed doses, uniformly delivered in a few minutes in multiple fractions in EBRT. The BED accounts not only for the absorbed dose but also for the dose rate, when assessing the tissue response to the radiation injury. More recently, the LQM has been reformulated to model therapies with continuously variable dose rate, and possibly non-uniform absorbed dose distribution, such as RE. Thus, for two different radiation modalities with the same BED, the same biological effect is expected to occur, provided to be able to evaluate the BED with enough accuracy.

The principal equations of the radiobiological method are here summarized:

(1) $$\text{ln }\left(\text{SF}\right)=-\alpha \text{ BED}$$

(2) $$\text{for EBRT:}\;\text{BED}=D_{\text{EBRT}}\left(1+\frac{D_{\text{EBRT}}\text{/}N}{\alpha \text{/}\beta }\right)$$

(3) $$\begin{array}{cccc} \text{for RE:}\;\text{BED} & =D_{\text{RE}}\left(1+\frac{D_{\text{RE}}\cdot \lambda_{\text{eff}}}{\left(\mu_{\text{rep}}+\lambda_{\text{eff}}\right)\cdot \alpha \text{/}\beta }\right)\; & & \;\\ & \;\text{or }D_{\text{RE}}\left(1+\frac{D_{\text{RE}}\cdot T_{\text{rep}}}{\left(T_{\text{rep}}+T_{\text{eff}}\right)\cdot \alpha \text{/}\beta }\right) \end{array}$$

where SF is the fraction of cells surviving after irradiation, α/β gives the curvature of the survival curve and relates the intrinsic radiosensitivity α and the potential sparing capacity (β), *D*_EBRT_ is the absorbed dose delivered with EBRT in *N* fractions (absorbed dose per fraction: *D*_EBRT_/*N*), *D*_RE_ is the absorbed dose delivered with RE, λ_eff_ is the effective rate constant of a mono-exponential variation of the absorbed dose rate, as applies in RE, which accounts for a protracted radiation where repair of sub-lethal DNA damage can occur (in RE, λ_eff_ equals the physical decay constant of ^90^Y: λ_eff_ = λ_phys_ = 0.0108/h), μ is a mono-exponential repair constant of a single DNA strand damage, and *T*_eff_ (*T*_eff_ = *T*_phys_ = 64.2 h) and *T*_rep_ are the halftimes for ^90^Y decay and repair damage.

The radiobiological parameters included in Eqs 1–3 are specific for the tissues and the effects (typically, α/β is assumed to be 2.5 Gy for the normal tissue and 10 Gy for the *T*. *T*_rep_ = 2.5 h for NL (μ_rep_ = 0.28/h), 1.5 h for *T* (μ_rep_ = 0.53/h)).

In case of spatial absorbed dose non-uniformity, the equivalent uniform BED (EUBED) has been used for *T* and organs with parallel structure to represent the uniform biological absorbed dose, which would produce the same number of surviving cells (64). It can be calculated to assess the possible radiobiological effect, according to the following equation:

(4) $$\text{EUBED}=-\frac{1}{\alpha }\ln\left(\text{SF}\right)=-\frac{1}{\alpha }\ln\left(\int_{0}^{\infty }P\left(\text{BED}\right)\cdot e^{-\alpha \cdot \text{BED}}\text{dBED}\right)$$

where P(BED) represents the probability density function of BED, and exp(−αBED) expresses the fraction of surviving cells SF. This applies in case of *T* or in case of functional subunits of parallel organs, as the liver and the lungs (10, 65).

In some cases of RE, also the equivalent uniform dose (EUD) can be a useful parameter. It represents the absorbed dose given uniformly that will lead to the same effect as the actual non-uniform absorbed dose distribution, and can be extrapolated by solving the following equation:

(5) $$\text{EUBE}{\text{D}}_{\text{RE}}=\text{EU}{\text{D}}_{\text{RE}}\left(1+\frac{\lambda }{\left(\mu +\lambda \right)\cdot \alpha ∕\beta }\text{EU}{\text{D}}_{\text{RE}}\right)$$

This equation mirrors Eq 3 but accounts for non-uniformity.

Similarly, EUD for EBRT can be derived as the solution of:

(6) $$\text{EUBE}{\text{D}}_{\text{EBRT}}=\text{EU}{\text{D}}_{\text{EBRT}}\left(1+\frac{\text{EU}{\text{D}}_{\text{EBRT}}}{N\cdot \alpha ∕\beta \;}\right)$$

Concerning the risk of toxicity, the phenomenological curves of NTCP (66) derived from the EBRT experience can be considered. The expression proposed to fit the EBRT absorbed dose–response data for a uniform irradiation of the whole organ is:

(7) $$\text{NTCP}=\frac{1}{\sqrt{2\pi }}\int_{-\infty }^{\text{t}}\exp\left(-\frac{x^{2}}{2}\right)dx$$

(8) $$\text{with}\;t=\frac{D-\text{T}{\text{D}}_{50,5}}{m\cdot \text{T}{\text{D}}_{50,5}},$$

where *D* is the total absorbed dose, *m* is a parameter representing the steepness of the dose–effect curve, and TD_50,5_ is the absorbed dose value for which 50% of the population exhibited complications within 5 years for a uniform whole-organ irradiation.

An alternative definition of EUD was proposed in EBRT. When the organ is irradiated with a non-uniform absorbed dose distribution, with fractions of volumes *v*_i_ uniformly irradiated with an absorbed dose *D*_i_ (identifying the series {*v*_i_, *D*_i_}), the effective volume method is applied in EBRT for reducing the DVH data to the unique parameter EUD (67). The NTCP associated to the non-uniform absorbed dose distribution can thus be calculated from Eqs 7 and 8 assuming that the whole organ is uniformly irradiated with an absorbed dose equal to EUD. The parameter EUD is defined as:

(9) $$\text{EUD}={\left(\sum_{\text{i}}D_{\text{i}}^{\frac{\text{1}}{\text{n}}}v_{\text{i}}\right)}^{\text{n}}$$

where *n* is the volume-effect parameter (e.g., *n* = 0 for serial organs, 0 < *n* < 1 for serial-parallel organs).

In order to apply the same model in case of an hypothetical uniform RE irradiation, the BED values from RE need to be converted into the equivalent dose at 2 Gy/fraction (EQD2), the standard treatment of EBRT, according to Eq 10:

(10) $$\text{EQD}2=\frac{\text{BE}{\text{D}}_{\text{RE}}\cdot \alpha ∕\beta \;}{2+\alpha ∕\beta \;}$$

Similarly, in case of non-uniformity, the {*v_i_*, BED_i_} series derived from molecular radio-therapy (MRT) can be converted into a series {(*v*_i_, EQD_i_)}, which are the input for Eq 9 to derive EUD, and then apply Eqs 7 and 8.

As regards *T*, a generalized expression of BED includes the effect of repopulation that occurs during treatment and wasting some of the delivered absorbed dose (68). Considering an exponential clonogen proliferation with doubling time *T*_av_ and a time of treatment *T*, the Eq. 3 becomes:

(11) $$\text{BE}{\text{D}}_{\text{tumor}}=D_{\text{RE}}\left(1+\frac{D_{\text{RE}}\cdot T_{\text{rep}}}{\left(T_{\text{rep}}+T_{\text{eff}}\right)\cdot \alpha ∕\beta \;}\right)-\frac{\text{ln}2}{\alpha T_{\text{av}}}T$$

Finally, for a certain *T* BED (or EUBED in case of non-uniform absorbed dose distribution), the TCP is defined (69) for an initial number of clonogenic cells *N*_0_ as:

(12) $$\text{TCP}=\exp\left(-N_{0}\cdot \text{SF}\right)=\exp\left(-N_{0}\cdot \exp\left(-\alpha \cdot \text{BED}\right)\right)$$

## Comparing RE and EBRT

Few models of TCP and NTCP have been reported in literature that are able to predict the clinical outcome of EBRT, confirming that a correlation is possible when based on the LQM. Considering the BED concept as possible rationale for RE planning, the equations of the previous paragraph are the simplest way to convert the absorbed doses from RE to EBRT, and *vice versa*. Moreover, it is possible to follow the EBRT imprint, comprising DVH, dose–volume reduction, EUBED, and/or EUD evaluations. This conversion could allow to derive estimates of prescribed limits to fulfill in RE based on EBRT studies, such as the effects of single lobe (or segment) versus whole liver irradiation, and the possible consequences of concurrent chemotherapy or re-treatment (15). However, the problem is complex due to the liver structure and the different irradiation conditions. Two different end points are under study, since in EBRT the centrilobular vein is the critical target, while in RE the portal tracts have different irradiation mechanism. Moreover, RE deeply differ if using glass rather than resin microspheres, as described in Section “Side effects to the liver.” From this point, the irradiation conditions of resin spheres are more similar to EBRT, resulting in more uniform dose deposition.

A purely mathematical example of tolerance doses derived from EBRT is reported in Table 3 assuming uniform dose distribution to the liver and lungs. This could be taken as starting point for proper phase I absorbed dose-escalation studies, which were never done. The following table was extrapolated using a set of radiobiological parameters taken from EBRT, while this applicability should be experimentally confirmed for RE. It is not directly applicable to glass microspheres. For whole liver irradiation, the same biological effect caused by an absorbed dose of 30 Gy (TD_5/5_) with EBRT could correspond to a BED of 54 Gy and to an absorbed dose of 35 Gy uniformly released by resin sphere RE.

**Table 3:** **Mathematical example of tolerance doses derived from EBRT assuming a uniform dose distribution to liver and lungs**.

	Treated volume	TD_5/5_ (Gy) EBRT	TD_50/5_ (Gy) EBRT	BED_5/5_ (Gy) EBRT = RE	BED_50/5_ (Gy) EBRT = RE	D_5/5_ (Gy) RE	D_50/5_ (Gy) RE
Liver	3/3	30	40	54	72	35	44
	2/3	35	45	63	81	40	47
	1/3	50	55	90	99	51	55
Lungs	3/3	17.5	24.5	29	41	23	30
	2/3	30	40	50	67	35	43
	1/3	45	65	75	108	47	62

*Liver absorbed doses from EBRT (70) converted to BED (values are the same EBRT and RE) and extrapolated for RE assuming α/ β = 2.5 Gy (liver), 3 Gy (lungs), *T*_rep_ = 2.5 h*.

A second aspect is that smaller volumes of a “parallel organ” (e.g., liver or lungs) can tolerate higher absorbed doses (volume effect). In fact, Emami et al. (70) derived tolerance values producing a risk of 5% for partial irradiation of the liver, resulting in 35 and 50 Gy for an irradiation volume equal to 2/3 and 1/3 of the whole organ, respectively. This suggests that lobar or segmental RE treatments can deliver higher absorbed doses when compared to absorbed doses delivered to the whole liver. Moreover, the lower NL is the volume treated, the higher is the tolerability. According to Dawson et al., irradiating less than 25% of the liver volume allows whichever dosage (71). This was clinically verified by Rhee et al. in super-selective treatment with glass spheres with a median dose of 348 Gy to liver segment (72). RE with resin spheres involving only the right lobe or the left lobe could be roughly compared to an EBRT irradiation of 2/3 and 1/3 of the whole liver, respectively.

A third issue is represented by the different tolerance according to the basal liver status (liver function). Unfortunately, it is rare that a patient is planned for RE without previous treatments or underlying disease. Metastatic patients planned for RE usually have already received chemotherapy, while HCC is usually superimposed to liver chyrrosis. Patients naïve to RE or with a better liver status should have a higher tolerability as compared to patients after several chemotherapy treatments or with underlying disease. The EBRT experience shows different NTCP curves according to different basal status (73). This has been observed to apply also to RE clinical experience (59). A more detailed comparison of EBRT data can be found in the review of Chiesa et al. (12). After RE with glass spheres, the same group reported an increased liver decompensation rate according to the worsening of Basal Child (13).

A fourth issue concerns the cases of re-treatment. In EBRT, the cumulative absorbed dose to the well-known limits for toxicity. On the contrary, the literature reports several examples of re-treatment in RE, where dose limits were applied to each single administration, but that were overcome cumulatively. Both producer’s indications do not mention the problem of re-treatment, meaning that the same BSA or 120 Gy prescription could be applied repeatedly. This could explain the toxicity reported by some authors after multiple RE treatments (53, 74). The problem of safety re-treatment lacks of specific published data. From the radiobiological point of view, multiple administrations with reduced parenchyma adsorbed dose (e.g., 2 × 20 Gy) theoretically result in lower BED, i.e., in lower toxicity, than a higher single dose of 40 Gy in a single administration (15). This aspect can be explained by using LQM and dose-rate effect, well known in EBRT and in some radionuclide therapies (10, 11). The cost–benefit ratio of this argument deserves attention and clinical validation and should consider the life expectancy of patients and the complexity of repeated intra-arterial administrations.

## Dose–Effects Correlations

The present section proposes a comprehensive overview of the literature as regards the possible correlations between absorbed doses and radiation-induced effects, on normal tissues and *T*.

### Side effects

The principal risks associated to RE are due to an excessive irradiation to NL and to an extra-hepatic shunt (observed in a low percentage of patients). If an extra-hepatic shunt exists, relevant toxicities, such as radiation pneumonitis, gastric/duodenal ulceration, and radiation cholecystitis can occur (5). LS is observed especially in HCC patients, who need quantitative evaluation of activity driven to lungs. Gastroduodenal complications occur in less than 5% of cases and can be prevented by careful evaluation of ^99m^Tc-MAA-SPECT images. A potential risk of red marrow irradiation related to possible free ^90^Y is due to its natural tropism for the bone. About hematological toxicity, the review of Riaz et al. (5) indicates ~25% lymphocytes reduction in the majority of treated patients. G3 or G4 lymphopenia was reported by Mazzaferro et al. (75) and by Hilgard et al. (76) in ~10% of HCC patients, but the exact origin is still unclear.

To date, the risk of complications induced by RE is acceptable when patients are adequately selected and the procedure of microsphere injection is carefully operated. Nevertheless, threshold absorbed doses avoiding toxicity are still not definitely identified, being neither dosimetry nor NTCP modeling systematically applied, or properly reported. For instance, the studies on glass spheres have usually provided absorbed dose values averaged on the injected portion of liver (without differentiating between NL and *T*), losing the information about the actual tolerability of the liver parenchyma and of the irradiation required to control the tumor. Using BSA method with resin spheres, the absorbed doses to NL vary in a wide range [median 36 Gy, range: 6–78 Gy (16)]. Moreover, an average dose that assumes uniform activity does not reflect the actual microsphere distribution (36). These drawbacks have been recently highlighted and works reporting dosimetry information, as well as the quality of dosimetric evaluation, has consistently increased. Studies at the microscopic level have offered complementary information that allow to better interpret the experimental findings [(1, 56, 60); section Activity Distribution at the Microscopic Level].

**Figure 1:**
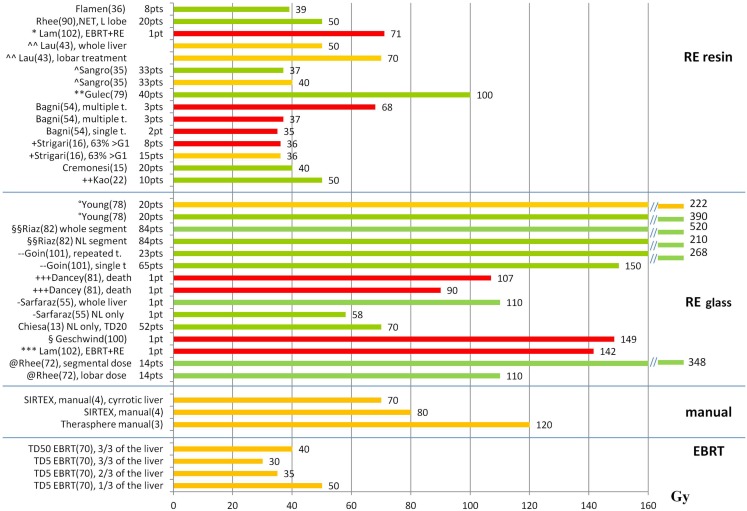
**Liver absorbed doses (Gy) and tolerability**. The graph shows the liver absorbed doses (Gy) reported in the literature with information about the associated liver tolerability. Red bars represent liver toxicity with fatal event (death); orange bars represent the threshold for observed toxicity or the limit recommended by the author; green bars represent tolerated absorbed doses. References are reported in parenthesis after the name of the first author. t., treatment; *(77) patient with previous EBRT (21 Gy) and RE with 71 Gy, ^∧∧^(42) recommendation based on a review of the literature, ^∧^(59) primary + mets, WL treatment, **(78) primary + mets, WL treatment, + (16) HCC, WL (48%) and L (52%), mean dose to WL for REILD > G1: 6–78 Gy. The bar represents the median value of the interval (36 Gy), ++(22) WL treatment,°(79) O-I, HCC, multiple treatments, §§(80) HCC, segmental treatments, - -(81) HCC, WL, and lobar treatments. No use of REILD toxicity score, +++(82) HCC, 9 O-I, 11 O-II, no distinction between tumor and NL dose, -(54) mean NL dose = 58 Gy, §(83) patient receiving RE to the right lobe (139 Gy) and the left lobe (158 Gy). The bar represents the mean value between the right and left lobe, ***(77) patient with previous EBRT (23 Gy) and RE to the right (111 Gy) and the left (172 Gy) lobe. The bar represents the mean value between the right and left lobe, @(84) HCC, segmental treatments, superselective

**Figure 2:**
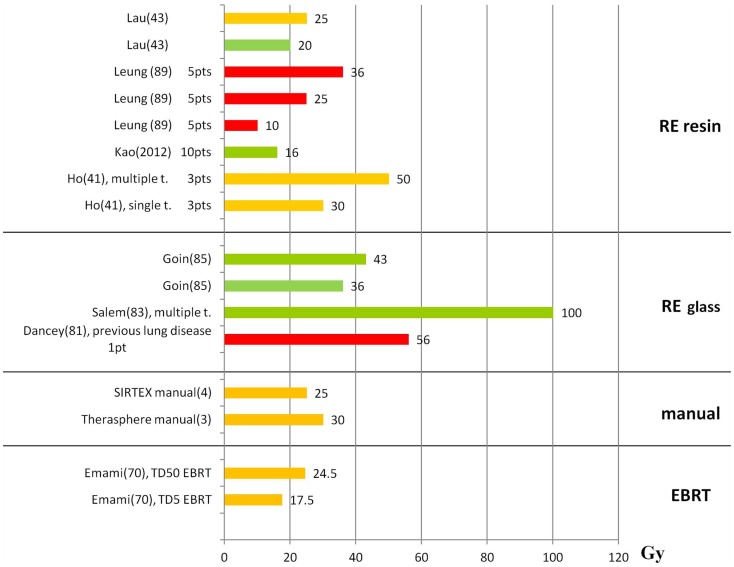
**Lung absorbed doses (Gy) and tolerability**. The graph shows the lung absorbed doses (Gy) reported in the literature, with information about the associated lung tolerability. The absorbed doses taken from the literature are reported although these are derived without including the attenuation correction. Absorbed dose values should be rescaled by an average factor of 0.6 (12). Red bars represent radiation-induced pneumonitis leading to death; orange bars represent the threshold for observed radiation-induced pneumonitis or the limit recommended by the author; green bars represent tolerated absorbed doses. The references are reported in parenthesis after the name of the first author.

Figures 1 and 2 summarize the correlations between absorbed doses and side effects to the liver and the lungs, highlighting tolerated absorbed doses (green bars), limits for toxicity or recommendations (orange bars), and manifested toxicity (red bars). Table 4 reports the fatal events documented in the literature due to radiation liver toxicity (Table 4A) and radiation pneumonitis (Table 4B).

**Table 4:** **(A) Fatal events due to liver failure; (B) Fatal events due to radiation pneumonitis**.

Reference	Type of spheres	Method for activity determination	No. of patients with adverse events leading to death	HCC or metastases	Notes
**(A)**					
Lau et al. (102)	Resin	Empirical	4% (3/71)	HCC	Liver failure but not RILD
Geschwind et al. (83)	Glass	100 Gy to whole liver; 135–150 Gy to whole liver	1% (1/80)	HCC	139 Gy to right lobe; 158 Gy left lobe
Goin et al. (81)	Glass	50–150 Gy to the whole liver or the lobe treated	5% (6/121)	HCC	In practice: 130 (34–268) Gy
Dancey et al. (82)	Glass	100 Gy to whole liver	9% (2/22)	HCC	Absorbed doses of 90 and 107 Gy; whole liver treatment (range given: 46–145 Gy)
Sangro et al. (101)	Resin	BSA; <60 Gy to NL	8% (2/24)	HCC	53 Gy (2.43 GBq) and 46 Gy (2.04 GBq)
Kennedy et al. (24)	Resin	empirical	4.1% (28/515)	HCC and metastasis	In the same center using the Empirical method
		BSA	1.5% (7/515)		
Strigari et al. (16)	Resin	BSA	11% (8/73)	HCC	(plus 15/73 pts with hepatic coma)
Bagni et al. (53)	Resin	BSA (1.6, 1.68, 1.3 + 1.3, 1.85 + 1.5, 1.85 + 1.5 GBq)	4% (5/135)	Metastasis	Mean absorbed dose to the liver: 35, 38 Gy (single treatment); 37–68 Gy cumulative
Lam et al. (77)	Resin	BSA(1.5 GBq)	3% (1/31)	Metastasis	21 Gy from EBRT and 71 Gy from RE
	Glass	120 Gy to the treated liver (7.3 GBq)	3% (1/31)	HCC	23 Gy from EBRT and 172 Gy (left lobe) + 111 Gy (right lobe) from RE
**(B)**					
Leung (89)	Not specified	Not specified	3/80 pts	HCC	LS within 13–46%, absorbed doses 10, 25, and 25 Gy without attenuation correction, corresponding to ∼6, 15, and 15 Gy with attenuation correction
Dancey (82)	Glass	100 Gy to the whole liver (NL+T)	1/22 pts	HCC	LSF 39%; absorbed dose of 56 Gy without attenuation correction, corresponding to ∼34 Gy with attenuation correction; previous lung chronic disease and lung embolism

*The absorbed doses values in the “Notes” column report the specific values given to patients who manifested a fatal event*.

#### Side effects to the liver

Some authors have distinguished between the classic radiation-induced liver disease (RILD) associated to EBRT, and the occurrence of REILD (59), the two having different profiles. The manifestation of RILD occurs typically 2–24 weeks after EBRT. It is dominated by vascular injury to the central vein, which is the most radiosensitive tissue in the hepatic microanatomy, characterized by a wall thickening that leads to VOD. There is the development of portal hypertension, ascites, and altered liver function tests, in particular an increase of alkaline phosphatize level, while liver enzymes and total bilirubin level may change only slightly (85, 86). Instead, REILD manifests typically within 2 months after RE and can damage mainly the portal tracts. A subacute and chronic portal triaditis could represent the effect of higher absorbed doses in the portal triads. The common clinical effects are ascites, jaundice, total bilirubin increase, increase in alkaline phosphatase and GGT, and splenomegaly at various degrees, which might suggest subclinical or low-grade portal hypertension (1, 12). The distinction between RILD (injury to the central vein) and REILD (injury to the portal tracts) compares well with the absorbed dose distribution at the microscopic level derived by mathematical modeling of the microspheres within the vessels (1, 60) (see section Activity Distribution at the Microscopic Level).

Liver toxicity is evaluated by assessment of enzymes and metabolites, i.e., ALT, AST, alkaline phosphatize, albumin, bilirubin, INR, or clinical symptoms (clinically detectable ascites, bleeding from esophageal varices, and encephalopathy). In patients with HCC, it is difficult to assess whether liver toxicity is due to worsening of hepatic cirrhosis, to disease progression or to RE. Timing is the argument generally adopted to consider a liver adverse event as treatment related or not. Events within 3 or 6 months are usually considered treatment related, depending on the authors. There is a lack of consensus about the exact liver toxicity definition, which encumbers data comparison. Mazzaferro et al. (75) introduced an *ad hoc* definition of liver decompensation, with a wide acceptance window (a cutoff of 6 months after treatment, including reversible adverse events), while Garin et al. (14) choose a 3 months cutoff, including only irreversible events and excluding cases attributable to *T* progression or portal vein presence. This resulted, for the same kind of HCC patients treated in the same way, in a liver decompensation incidence of 36.5% for the first group, while 9.5% for the second.

When reporting dose–effects to the liver, distinction needs to be made between resin and glass spheres, as absorbed doses values are not directly comparable (Table 2): for resin spheres, the mean absorbed dose to the NL is generally reported, while for the glass spheres the mean absorbed dose to the whole liver or treated lobe is usually expressed. As can be seen from viewing Figures 1 and 2, tolerance levels for glass spheres are higher in terms of grays.

##### Liver toxicity after resin spheres

Kennedy et al. (24) presented the results of 680 treatments on 515 patients. The median prescribed activities were 2.0 ± 0.4 GBq (empirical method), 1.6 ± 0.5 GBq (BSA method), and 1.1 ± 0.6 GBq (actually delivered). REILD occurred in 28 treatments (4%) and lead to death. Of these, in 21 of a single center, the activity to be administered was decided by the empiric method, and 20 were a bilobar approach. Unfortunately, the absorbed doses to NL are not available. According to the authors, REILD significantly correlated with the administered activity, thus indirectly with the liver absorbed dose. The high risk of death with method (i) lead to the recommendation to avoid the empirical method and whole liver treatments (5).

Sangro et al. (59) observed an increased toxicity in patients receiving an absorbed dose to NL of 37 ± 12 Gy versus 26 ± 12 Gy (^99m^Tc-MAA, partition model) (Figure 1). VOD was observed only in patients who received chemotherapy. The incidence of REILD was associated with young age (<55 years), diffuse disease, bilobar treatment, previous chemotherapy and/or pre-existing altered liver function, bilirubin level, and administered activity. The results indicated as threshold for REILD the mean absorbed dose of 40 Gy to NL.

The use of the LQM model as rationale of RE was proposed by Cremonesi et al. (15), who applied the limit of 40 Gy to NL in 20 patients with liver metastasis treated with total liver approach. The threshold value was extrapolated from the TD_5,5_ of EBRT, with proper conversion to RE by the BED concept, using a limit of 35 Gy (BED = 54 Gy, EQ2 = TD_5,5_ = 30 Gy) in a few patients, followed by an absorbed dose escalation to 40 Gy (BED = 64 Gy). No toxicity was observed apart from transient elevation of liver enzymes. The authors suggested the use of the LQM also as theoretical basis to evaluate the risk/benefit balance of re-treatment or bilobar approaches, which are empirically applied instead (72, 79, 80, 86).

Out for 135 patients, Bagni et al. (53) reported 5 cases (4%) of liver failure that led to death 3–4 months after RE. The activity ranged from 1.3 to 1.85 GBq (BSA method). Simulation with ^99m^Tc-MAA was not performed, but post-therapeutical ^90^Y-PET images showed diffused radioactivity in the liver and reduced tumor uptake. Voxel dosimetry was performed. Two patients received a single treatment with mean absorbed doses to NL of 34.5 Gy (EQ2 = 30 Gy, BED = 54 Gy) and 38.5 Gy (EQD2 = 33 Gy, BED = 61 Gy) assuming α/β = 2.5 Gy. The other three patients underwent two treatments with cumulative mean absorbed doses ranging from 37 to 68 Gy (EQD2: 31.8–76.5 Gy, BED: 57.3–137.7 Gy). Details about the clinical status of the patients were not reported, but the absorbed doses received also in the first treatment adhere to the limits extrapolated from EBRT. These cases with resin spheres showing – at least from imaging – nearly uniform irradiation of NL, are more closely reproducing the uniform irradiation of EBRT.

Strigari et al. (16) applied to a nuclear medicine treatment the radiobiological methods used in EBRT, pointing to the methodology that should be used to describe the effects and to plan any radiation treatment nowadays. The authors analyzed 73 HCC patients Voxel dosimetry was retrospectively assessed from Bremsstrahlung images. They found that the mean absorbed dose was a predictor for liver failure and a modified Lyman–Burman–Kutcher model was applied to obtain a fitted NTCP curve with toxicity >G1 as end-point. The parameters describing the NTCP were a TD_50_ of 52 Gy (95%CI, 44–61 Gy) and a slope of NTCP versus dose of 0.28 Gy^−1^(95%CI, 0.18–0.60), assuming *n* = 1.

Di Dia et al. (51) applied the voxel dosimetry method to ^99m^Tc-MAA-SPECT images of 13 metastatic patients undergoing whole RE with resin spheres, with a prescribed mean absorbed dose of 40 Gy to NL (assuming an uniform distribution). No toxicity was observed, as expected. In principle, higher activities could have been administered according to the degree of non-uniformity highlighted by the voxel dosimetry. Average doses, BED, EUBED, and EUD, were calculated for several α and α/β values. EUD was considered as possible new constraint, in place of the mean absorbed dose. However, it was found that EUD is notably influenced by the value of α. The authors conclude that the α parameter needs to be identified for NL and the specific tumors in order to apply radiobiological quantities for RE protocols.

Lau et al. integrated the clinical experience from literature in a review to guide patient selection and activity planning for RE with resin microspheres (42). In order to minimize fatal events, the authors recommend 50 Gy as maximum limit in whole treatments and 70 Gy to the NL lobe treated in lobar approach. Kao et al. (22) did not find toxicity fulfilling these constraints.

Petitguillaume et al. (87) applied for the first time direct Personalized Monte Carlo Dosimetry (PMCD) to resin sphere treatment. CT images created patient-specific voxel phantoms using the OEDIPE software. ^99m^Tc-MAA SPECT images of 10 patients were combined to calculate absorbed dose at voxel level with MCNPX Monte Carlo code. Activity prescription from PMCD and partition model were compared, with or without including DVH criteria. The use of PMCD resulted in higher administrable activity than the partition model calculation. The allowed increase is on the average of 27% if mean dose constraints are considered, and of 40% if DVH criteria are adopted.

##### Liver toxicity after glass spheres

Dancey et al. (82) treated 22 HCC patients (9 Okuda stage I, and 11 Okuda stage II) in a whole liver approach. The median (range) absorbed dose to the whole liver was 104 (46–145) Gy, and to the lungs was 13.0 (1.8–56.5) Gy [with LF: 6.4 (1–39%)]. Two patients were treated a second time, resulting in total liver doses of 100 and 209 Gy and lung doses of 43 and 36 Gy, respectively. Serious adverse events (severe, life threatening, and death) occurred in 14 patients (63%), including three deaths. The authors admit that the lack of detailed liver dosimetry with distinction between *T* and NT parenchyma is a major limitation of their study.

Sarfaraz et al. (54) first investigated the voxel activity distribution in one treated patient. The absorbed dose distribution was retrospectively analyzed by ^99m^Tc-MAA-SPECT images, with isodose curves and DVHs derived for *T* and liver. The mean *T* and NL doses were 163 and 58 Gy. No radiation hepatitis was reported for this patient, in agreement with the reduced amount of liver irradiated with more than 110 Gy and with the use of glass spheres, 58 Gy is in fact much higher than the tolerance observed in resin spheres and in EBRT.

Goin et al. (81) analyzed a group of 88 patients affected by HCC. The results indicated that pre-treatment bilirubin and liver absorbed dose were the most statistically significant factors associated with an increased risk of liver toxicity. A limit of 150 Gy for a single administration and of 268 Gy for a repeated RE were considered as tolerated and reversible. The authors concluded that no cases of serious RILD occurred, being the bilirubin increase, and not the alkaline phosphatase, the mark accompanying ascites in 33% of patients. However, they did not consider the definition of REILD that possibly occurred.

Rhee et al. (72, 84) used CT angiography (CTA) in 14 patients with unresectable HCC to super-selectively administer in liver segments and to evaluate tumor absorbed doses. Activity was planned in order to deliver 120 Gy to the target lobe. Estimated absorbed doses to the lobe (before CTA) (100 ± 43 Gy, range: 35–169 Gy) were significantly lower than the actual absorbed doses to the target segmental liver volume (after CTA) retrospectively calculated (348 ± 204 Gy, range: 105–857 Gy). Changes in serum bilirubin level were statistically significant within normal levels and were not clinically relevant. Thirteen of 14 patients had no change in Child-Pugh class. This study supports that irradiation of a small organ fraction gives very limited toxicity, enhancing that the well-known liver volume effect in EBRT also applies in RE (see also section Comparing RE and EBRT).

The relationship between cumulative lobar radiation dose and liver toxicities in case of multiple RE procedures has been investigated by Young et al. (79). Forty-one patients affected by HCC and classified according to the Okuda stage I (O-I, 20 patients) and stage II [(O-II), 21 patients] disease were enrolled. The O-I group received more treatments than the O-II group, and a higher cumulative absorbed dose to the lobes, with average (range) 247 (88–482) Gy versus 198 (51–361) Gy. Toxicity was observed in only 16% of patients. O-I patients received a greater cumulative dose than O-II patients before liver function alteration: 390 versus 196 Gy, confirming different tolerance for different initial conditions (section Comparing RE and EBRT). For O-I patients, a higher cumulative absorbed dose was associated with occurrence of one or more toxicities: 222 Gy (no toxicities) versus 390 Gy (≥1 toxicity), confirming that liver toxicities increase with increasing cumulative radiation dose. It is worthy to note that the O-I group manifesting greater tolerability received the cumulative therapy in a higher number of treatments (2.65 versus 2.24 treatments in average), possibly shifting the tolerance toward higher absorbed dose values (see also section Comparing RE and EBRT).

Riaz et al. (80) further applied the segmental RE approach to 84 selected HCC patients. Activity was planned to deliver an absorbed dose of 120 Gy to the lobe. The segment absorbed dose was estimated with the hypothesis of uniform activity, but also with a revised method based on the iodinated contrast medium distribution (subjective assessed by the radiologist), in the attempt to assess a T/NL ratio. The median dose to the segment was 521 Gy (95%CI: 404–645 Gy) by the usual method, while it was 210 Gy (95%CI: 107–270 Gy) with the revised method. The results highlighted that the T/NL uptake ratio has a great impact on evaluation, remarking the importance of separate *T* and NL dosimetric evaluation. G3 and G4 toxicity rate was extremely low, confirming the volume effect in RE (see also section Comparing RE and EBRT).

**Figure 3:**
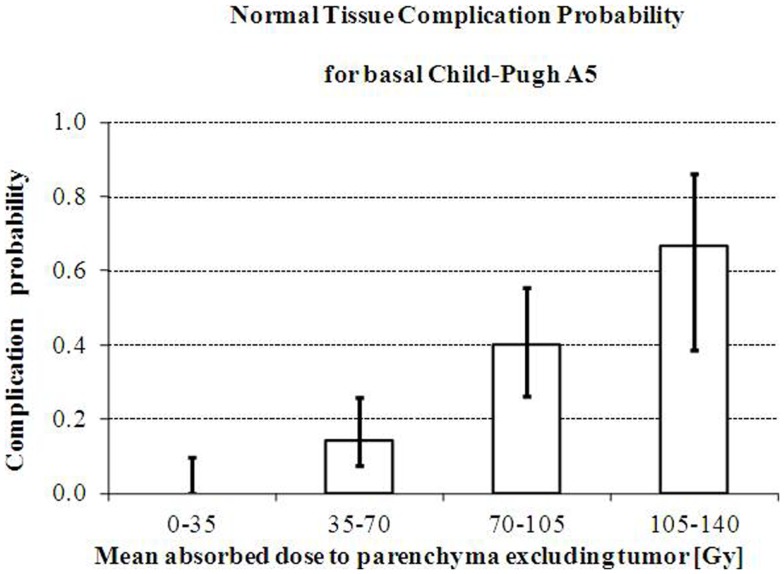
**Increase of risk of liver decompensation with mean absorbed dose**. Increase of the observed risk of liver decompensation with whole parenchyma mean absorbed dose in intermediate/advanced HCC patients with Basal Child-Pugh A5 treated with glass microspheres. Reprinted with permission by Minerva Medica from *Quarterly Journal of Nuclear Medicine Molecular Imaging* (13).

Chiesa et al. (13) retrospectively evaluated on ^99m^Tc-MAA SPECT images the *T* and NL absorbed doses of 52 HCC patients lobarly injected according to the prescription of 120 Gy (75). Liver decompensation was defined according to Mazzaferro et al. (75). Basal Child-Pugh strongly affected the toxicity incidence: 22% for A5, 57% for A6, and 89% for B7 patients (univariate analysis). In Child-Pugh A5 patients, absorbed dose averaged on NL was 90 Gy (liver decompensation) versus 58 Gy (non-toxic treatments). The experimental NTCP histogram as a function of NL mean-absorbed dose is shown in Figure 3. A limit of about 70 Gy for the mean absorbed dose to parenchyma can be assumed for Child A5 patients treatment planning, corresponding to a 14% risk of liver decompensation. This result is applicable only with glass spheres decay interval of 3.75 days.

#### Side effects to the lungs

Significant shunting to the lungs is rather rare and avoidable by appropriate patient exclusion criteria. It can lead to progressive pulmonary insufficiency, with pulmonary fibrosis, and ultimately radiation pneumonitis (88). Some authors report the acceptable limit by means of LS percentage, others report the LS and absorbed dose limits. Of relevance, the lung absorbed dose evaluations that are reported in this paragraph and in Figure 2 are overestimated, because authors usually do not apply the attenuation correction. The proper values – also for possible comparison with EBRT – should be rescaled by an average factor of 0.6 (12). E.g., the limits of 30 and 50 Gy (40, 89) obtained with resin spheres from non-attenuation corrected images, should be actually 18 and 30 Gy, much closer to the TD values from EBRT (TD_5_ = 17.5 Gy; TD_50,5_ = 24.5 Gy) (70).

##### Resin spheres

Leung et al. (89) described 5/80 cases of radiation pneumonitis, with 3 patients who died from respiratory failure. The LS of these patients ranged from 13 to 46%, with associated absorbed doses from 10 to 36 Gy. Recommendations of LS < 13% (corresponding to 19 Gy for 3 GBq of microspheres injected) were given, as 55% of the patients having this LS developed toxicity, which was absent in patients with LS < 13%. Furthermore, 19 Gy was indicated as the lower value to cause radiation pneumonitis.

From the same group, Ho et al. (40) suggested a LS of 20% and an absorbed dose of 30 Gy as limits for risk of radiation pneumonitis in a single treatment, and a cumulative absorbed dose of 50 Gy not to be exceeded in repeated treatments.

In a recent paper regarding resin spheres, Lau et al. (42) recommended that lung dose remains < 20 Gy, and never exceeds 25 Gy (a more cautious approach than 30–50 Gy limits or LS < 15–30%).

Kao et al. (22) did not find any significant toxicity in 10 patients receiving mean lung absorbed doses lower than 16 Gy.

##### Glass spheres

Dancey et al. (82) report the death of one patient for radiation-induced pneumonitis (6 weeks after RE), with a LS of 39% and a lung absorbed dose of 56 Gy. In this single case of death from radiation-induced pneumonitis, the patient had a chronic lung disease, and a pulmonary embolism occurred 4 weeks before RE. Instead two patients who received a dose to the lungs > 30 Gy (36 and 43 Gy) did not develop any serious adverse pulmonary events (86).

In the study by Salem et al. (88), 58 patients who underwent RE received a lung absorbed dose > 30 Gy in single treatment and 50 Gy cumulatively without developing radiation pneumonia. Only 10/53 patients exhibited G1 lung toxicity. Absorbed doses to the lungs higher than 100 Gy were tolerated without any adverse effect. The authors conclude that the most applied limits (30 Gy single, 50 Gy multiple), should be revised and that the model – based on simulation by MAA, assuming uniform activity distribution in the lungs, with same limits for resin and glass microspheres – might not be adequate.

Literature on EBRT has shown that the incidence of radiation pneumonitis better correlates with the volume receiving a certain absorbed dose (e.g., V20 or V30 as lung volume receiving at least 20 or 30 Gy) rather than the mean lung absorbed dose (90–92). A better comparison with the EBRT models would require an accurate evaluation of the activity biodistribution in the lung vasculature, incorporating the possible differences of resin and glass microspheres. The microspheres distribution in the lungs is probably uneven, with a preferential distribution to the lung bases and central parts, while a fraction is totally spared (7, 8). This may happen because the distribution is affected by gravity and by the blood flow (7). Moreover, the different number of particles could lead to a greater micro-embolic effect with resin as compared with glass microspheres also in the lungs (88, 89).

### Tumor response

**Figure 4:**
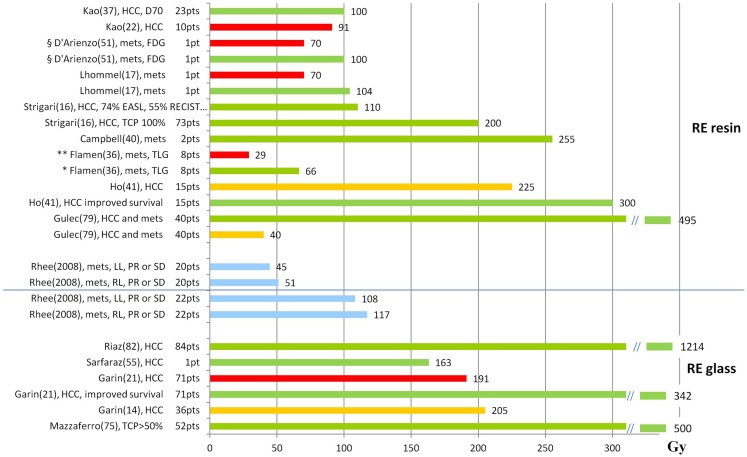
**Tumor absorbed doses (Gy) and response**. The graph shows the T absorbed doses (Gy) reported in the literature, with information about the associated response. Red bars represent progression, orange bars represent the threshold for response, green bars observed response, and blue bars specifically indicate PR (partial response) or SD (stable disease). References are reported in parenthesis after the name of the first author. mets, metastases; *response evaluation based on the variation of TLG in FDG examinations; **non-responders based on the variation of TLG in FDG examinations; §response evaluated based on the variation of SUV in FDG examinations.

As point of attention in reviewing published data is that different methods for tumor response evaluation have been considered, based on CT (RECIST, dimensional criteria), EASL (density), or PET parameters (e.g., SUV variation or total lesion glycolysis (TLG), defined as product of the mean lesion SUV and the volume of each lesion). The results available in the literature are summarized in Figure 4, where distinction is made among absorbed doses that are associated to response (green bars), thresholds for response (orange bars) and T progression (red bars). Blue bars specifically indicate PR (partial response) or SD (stable disease).

#### Resin spheres

Rhee et al. (84) evaluated the response of 42 patients who underwent RE using glass and resin microspheres. In all cases, the lobe but not the NL absorbed dose was evaluated. Using glass, a higher median absorbed dose was delivered to each lobe (right lobe 117 Gy, left lobe 108 Gy) than using resin (right 50.8 Gy, left 44.5 Gy). According to RECIST criteria, the 6-month disease control rate was similar with both microspheres (92% with glass and 94% with resin particles). This study gave the same clinical efficacy from different median absorbed doses, as a consequence of different number of microsphere per Gy and different non-uniformity of dose deposition on microscopic scale, as a consequence of different number of microsphere per Gy and different non-uniformity of dose deposition on microscopic scale.

Ho et al. (40) estimated the correlation between tumor absorbed doses and responses in a group of 71 patients affected by HCC. Repeated (two to five) treatments were given to 15 patients. Tumor doses were estimated by the partition model. The median absorbed dose at the first treatment was 225 Gy (range: 38–748 Gy), while cumulatively was 302 Gy (range: 83–1580). 37% of the patients showed a partial response at absorbed doses > 225 Gy, in comparison with 10% at absorbed doses < 225 Gy. For survival, with a cut-off value of 300 Gy, the median OS of 11 and 7 months was observed in patients above and below the cutoff, respectively, although the difference was not statistically significant.

**Figure 5:**
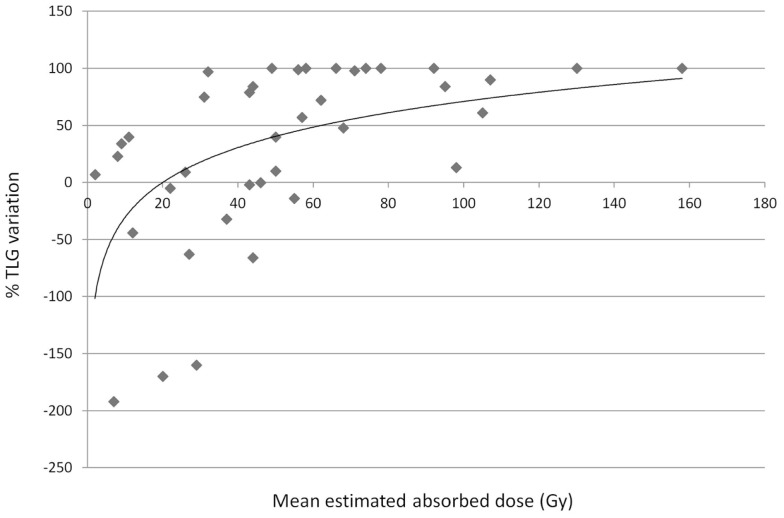
**Tumor response related to variation of the TLG with absorbed doses (Gy)**. *T* response by means of the TLG variation in FDG-PET examinations versus T absorbed dose. Regression analysis (*R*^2^ = 0.26). Errata corrige of previously published data by Flamen et al. (35). Data provided by the authors, personal communication.

Flamen et al. (35) made a retrospective dosimetry on ^99m^Tc-MAA–SPECT images of the PET response in 39 metastatic liver lesions from colorectal cancer in a group of 8 patients treated according to the BSA method (mean; range): 1.69 GBq; 1.33–2.04 GBq. The paper reports the absorbed doses of the lesions as a function of TLG variation (Figure 5), showing a clear trend, and a correlation coefficient of *R*^2^ = 0.26. This apparently low value is similar to others found in nuclear medicine dosimetry (93), and also in EBRT and might be attributed both to the mislocation of points on the abscissa (dosimetric inaccuracy) and in the ordinates (interpatient variability). Different response for the same dose may also be deeply influenced by the lesion dimension. The median (95% CI) absorbed dose was 29 (1–98) Gy and 66 (32–159) Gy in the poor (<50% TLG change) and the good responders (TLG change > 50%), respectively. From MAA-SPECT images using T/NL ratio of 1 as cut-off, a significant metabolic response was predicted with a sensitivity of 89%, a specificity of 65%, a positive predictive value of 71%, and a negative predictive value of 87%. Authors concluded that simulation with MAA can provide essential information for patients’ recruitment to RE, allowing to predict the metabolic response. The mean absorbed dose of the normal liver parenchyma was 39 Gy (32–48 Gy), without toxicity.

Campbell et al. (39) retrospectively analyzed 14 patients affected by metastases from colorectal cancer. The partition model was applied with a *T* to NL ratio arbitrary of 3:1 and a patient-specific ratio (^99m^Tc-MAA SPECT images). The response rate was evaluated comparing FDG-PET at baseline and 3 months after RE. There was a statistically different absorbed dose between the two methods. PET showed a mean decrease of 52% of total SUV in tumors. There was a linear correlation between absorbed dose and tumor response, but the tumor absorbed dose using a patient-specific method was more predictive (*R* = 0.65). Two good responses were observed at 255 and 317 Gy, for lesions of 5 and 13 g.

Strigari et al. (16) implemented a TCP model in RE analyzing the outcome of 73 HCC patients. Response was defined according to the RECIST and EASL criteria (94–96). The radiobiological parameters used to compute BED for HCC were α/β = 10 and *T*_rep_ = 1.5 h. Experimental TCP was fitted with the hypothesis of two different cellular radiosensitivity in *T*, obtaining two α-values (0.001 and 0.05 Gy). Note that these values are definitively lower than those obtained in EBRT (α = 0.01 ± 0.1/Gy) reported by Tai et al. (97) after *in vivo* EBRT. TCP curve indicated that all tumors with mean dose greater than 200 Gy showed a complete or partial response. At lower doses, a higher response rate was found using EASL rather than RECIST criteria, i.e., ^90^Y irradiation causes changing in *T* structure rather than its shrinking. With an average dose of 110 Gy to the tumor, complete or partial response was observed in 74 and 55% of patients according to the EASL and RECIST criteria, respectively.

The paper by Lhommel et al. (17) describes the new method for ^90^Y-PET imaging using the low branch of *e*-/*e*+ pair production in the ^90^Y decay (probability: 32 × 10^−6^) and a TOF scanner. The authors demonstrated that PET-based microsphere dosimetry is feasible and quantitatively accurate, and they applied the method in a patient treated with resin spheres. The absorbed dose distribution well correlated with tumor control, with good response in regions of high absorbed dose (average absorbed dose 104 Gy, maximum 241 Gy), and tumor progression in regions only partially targeted (average absorbed dose 29 Gy, maximum 70.6 Gy). The advantage of this post-therapy technique is the possibility to study the correlation based on the actual absorbed dose, i.e., bypassing the problem of correspondence between ^99m^Tc-MAA and microspheres, joined with the better PET spatial resolution with respect to SPECT. It is not useful however, to provide a treatment planning tool.

D’Arienzo et al. obtained high resolution post-RE ^90^Y images by a non-TOF PET/CT scanner provided with bismuth germanate (BGO) crystals (49, 50). The voxel dosimetry method was applied for the analysis of a patient with liver metastases, administered according to the BSA method (1.45 GBq) (50). The absorbed dose distribution map and DVHs for both *T* and NL, showed a wide heterogeneity. The mean absorbed dose to *T* was 139 Gy, but two areas were distinguished: a hot margin receiving an average absorbed dose of 287 Gy (range: 100–700 Gy) and a cold area with a necrotic core receiving an average absorbed dose of 70 Gy with a large proportion of voxels receiving < 50 Gy. At the FDG-PET control 6 months after RE, complete remission was observed in highly irradiated *T* areas, while progression was observed in the scarcely irradiated area, demonstrating correlation between absorbed dose and *T* response, and the role of non-uniformity of dose deposition. The authors conclude that an average radiation dose > 100 Gy may sterilize liver metastases.

Kao et al. (22) highlighted the power of predictive dosimetry in a study involving 10 patients with HCC and very different baseline disease characteristics. The authors introduce the “planning target volume” concept, adapted from EBRT, and state that “despite its popularity, the BSA method has a questionable radiobiological basis and is scientifically inferior to MIRD methodology.” The patients were therefore administered with resin microsphere using an artery-specific ^99m^Tc-MAA-SPECT/CT partition model. Median predicted absorbed doses were 106 Gy (95% CI, 105 ± 146 Gy) to *T*, 27 Gy (95% CI, 22 ± 33 Gy) to NL, and 2 Gy (95% CI, 1.3 ± 7.3 Gy) to lungs. The results showed that a 100% *T* response rate could be achieved with an absorbed dose of at least 91 Gy.

The same author (36) presented a study of 23 patients affected by HCC and metastases treated with activities based on predictive dosimetry as above. ^90^Y-PET images were acquired after RE and absorbed dose distributions obtained by voxel dosimetry. Dose–responses in tumor was analyzed considering the DVHs (^90^Y-PET) or the mean absorbed doses (^99m^Tc-MAA-SPECT) in correlation with follow-up imaging or clinical findings (98, 99). Interestingly, the concepts of D70 (the minimum absorbed dose delivered to 70% of the *T* volume) and V100 (the percentage *T* volume receiving ≥ 100 Gy) were proposed for *T* reporting from EBRT. D70 = 100 Gy was derived as a threshold for complete versus incomplete responses. Comparing the mean absorbed doses ^90^Y-PET and ^99m^Tc-MAA-SPECT imaging, a good correlation was found in 7 lesions under near-ideal dosimetric conditions (i.e., case of technically success, T/NL ratio ≥ 2, good activity coverage), with a relative error ranging from −2 to 13%.

#### Glass spheres

In a study previously discussed for toxicity, Riaz et al. (80) planned the activity in order to deliver 120 Gy to the lobe/segment, by the mono-compartmental method. Retrospectively, for comparison purposes, a method based on the iodinated contrast medium distribution was used to evaluate the absorbed dose to *T* and NL. Tumor received 1214 (961–1546) Gy. The response rate evaluated by WHO (dimension criteria) was 59% (after 7.2 months), and that evaluated by EASL (density criteria) was 81% (after 1.2 months). The study confirms the correspondence of high efficacy with high *T* absorbed doses. However, the difference of *T* absorbed doses between responding (EASL 1279 [986–1626] Gy) and non-responding (EASL (1118 [332–2139] Gy) lesions was not statistically significant, possibly because of the questionable method of assigning the *T*/NL uptake.

Sarfaraz et al. (100) used the standard method of 150 Gy to the treated lobe to establish the administered activity. They retrospectively analyzed 10 patients using the data from total body images (^99m^Tc-MAA scan) as input to the compartmental (partition) method. The activity distribution in the liver was found to be highly non-uniform, with different T/NL uptakes. For a typical patient, the absorbed doses to the *T* and NL were 402 and 118 Gy, respectively. The NL median dose with non-compartmental and compartmental model were statistically different (141 ± 12 Gy versus 117 ± 23 Gy). The authors pointed out the need to distinguish *T* to NL absorbed dose in order to avoid high risk of over- or under-dosage, and possibly to estimate the overall dose distribution.

In a subsequent paper (54), the same authors account the voxel activity distribution in a patient from ^99m^Tc-MAA SPECT images retrospectively analyzed, with isodose curves and DVHs derived for *T* and NL. The DVHs indicated that although the patient was treated to the nominal whole liver dose of 110 Gy, only 16% of NL and 83% of the *T* received a dose higher than 110 Gy. The mean *T* absorbed dose was 163 Gy.

In a retrospective study, Garin et al. (14) analyzed 36 patients with HCC. The activity was based on ^99m^Tc-SPECT/CT images in order to deliver an absorbed dose to the liver of 120 ± 20 Gy (without exceeding 30 Gy to lungs). The absorbed dose to the *T* (*D*_T_) and to NL were also determined. 69% of patients responded (EASL criteria). A multivariate analysis identified *D*_T_ as the only parameter associated with response, with the value of 205 Gy as threshold predictive of response, a sensitivity of 100%, and an accuracy of 91%. PFS and OS were 5.2 and 9 months when *D*_T_ < 205 Gy, whilst with *D*_T_ ≥ 205 Gy they were, respectively, 14 and 18 months. This study highlighted the utility of ^99m^Tc-MAA SPECT/CT dosimetry, in predicting both response and survival.

These preliminary results were confirmed in a larger cohort of 71 HCC patients (21), with the same threshold of *D*_T_ = 205 Gy for *T* response. The response rate was 79%, with a median *D*_T_ of 342 Gy and of 191 Gy for responding and non-responding *T*, respectively, with a statistically significant difference. Based on the predicted *D*_T_ and *D*_NL_, 17 patients underwent a boosted RE (increased injected activity with respect to 120 Gy prescription) that provided a good response rate (77%) without increased G3 liver toxicity. Median PFS and OS were only slightly different from the previous study. In 33 PVT patients, the median PFS and OS were 4.5 and 5 months when *D*_T_ < 205 Gy, whilst with *D*_T_ ≥ 205 Gy they were, respectively, 10 and 22 months. The authors remark the predictivity of ^99m^Tc-MAA SPECT/CT dosimetry on response and OS, and point out the possibility to adapt the treatment planning especially in patients with large *T*, e.g., intensifying the treatment without increasing liver toxicity (boosted RE concept).

In the retrospective dosimetry on ^99m^Tc-MAA SPECT images by Mazzaferro et al. (75), EASL lesion response (CR + PR) correlated with absorbed dose (Spearman’s *r* = 0.60; 95%CI, 0.41–0.74; *P* < 0.001). Lesions lacking of objective response received a median dose of 275 Gy, whereas responding tumors were found to absorb 490 Gy (*P* < 0.0001). An efficacy threshold of 500 Gy significantly predicted the observed objective response and limited to 20% the rate of non-responders (area under the curve = 0.78).

### Take home messages from dose–effects correlations

For the first time in nuclear medicine therapy, a dose–effect relation was indicated (observed), even though data are sparse and obtained with non-uniform methods. This is more evident for lesions, while a liver toxicitydose relation is reported in fewer papers. Several bias impede to find sharper dose thresholds: the heterogeneity of the end points considered, different basal liver conditions, the natural history of cirrhosis in HCC patients, the previous chemotherapy cycles for metastatic cases. Dosimetric methods are still not uniform but they are improving quickly. After the application in peptide radionuclide therapy (10), the radiobiological modeling (NTCP and TCP curves) inherited from EBRT has been applied to RE to describe the observed clinical data (16) and to plan treatments (13). The efforts made highlight several interesting issues that fit dosimetric and radiobiological perspectives. These are summarized for side effects and *T* responses in the following.

#### Side effects

- Administered activity alone does not appear to be a valid predictor of treatment safety compared to accurate estimation of absorbed dose (28 cases of death occurred although activities were not particularly high) (24). The empirical method has been excluded from recommendation. Despite its popularity, the BSA method represents a non-dosimetric approach and is radiobiologically questionable (22). The role of ^99m^Tc-MAA simulation is relevant, to avoid treatments with inappropriate activity distribution (14, 53).
- For resin spheres, the dosimetry with ^99m^Tc-MAA and the compartmental model was able to find out the indication for liver toxicity beyond 40 Gy to the whole liver (35, 101) – which is not far from EBRT limits (15). The BED_50_ of 93 Gy by Strigari et al. (16) in HCC patients is not so far from the BED_50_ of 72 Gy deduced from EBRT (70). In case of nearly uniform activity distribution, values for severe toxicity were comparable with those from EBRT (53).
- For glass spheres, authors acknowledge that the lack of distinction between *T* and NL is a major limitation (82, 100), as such distinction has a great impact on the evaluations derived (e.g., median absorbed dose to the liver segment of 520 versus 210 Gy by the usual glass method versus the revised method) (14, 21, 80). In any case, in glass spheres, increasing cumulative absorbed doses were found to increment liver toxicities (79). The NTCP reported in Figure 3 by Chiesa et al. is the first indication of augmented risk with absorbed dose averaged on the NL parenchyma for HCC patients. 70 Gy seems a good value to keep the incidence of liver decompensation below 20%.
- About lung tolerance, the present safety limits (dose < 30 Gy) were obtained for resin spheres on planar images non-corrected for attenuation. The correction leads this threshold close to the known value from EBRT (17.5 Gy). For glass spheres, the question is completely open.
- Dosimetry from post-RE images (Bremsstrahlung-SPECT but above all ^90^Y-PET) could improve the dose–effect correlations bypassing the problem of correspondence between MAA and microspheres (16, 49, 50, 53). The voxel dosimetry enriched the information about the degree of non-uniformity (50, 51, 53).
- The LQM was proposed as rationale to guide therapy decisions and absorbed dose-escalation studies involving re-treatment and partial liver irradiations (volume effects). The radiobiological methods used in EBRT to derive NTCP and TCP curves have been applied to RE to predict toxicity and response (16).
- The radiobiological parameters can consistently influence the radiobiological entities such as EUBED or EUD, and should be determined specifically for the tissues under investigation, in NL and different *T*. (51)
- The liver volume effect well known in EBRT has been confirmed also in RE, where the irradiation of a small organ fraction gave very limited toxicity (72, 80). A large irradiated volume with high dose is a risk factor (21).
- As in EBRT, the basal liver status deriving from underlying disease (cirrhosis) or concomitant or previous treatment (chemotherapy, TACE) results in markedly different dose tolerance (13, 59, 79). A systematic absorbed dose-escalation study would need the same conditions of the patients included (79).

#### Tumor response

- There is not a univocal evaluation criteria of *T* response (16, 35).
- In resin spheres, ^99m^Tc-MAA has provided essential information for patient recruitment to RE (35), preventing treatments without proper activity distribution (53). Lesion dosimetry correlated with metabolic response (17, 35, 50) as well as with radiologic response (14, 16, 39, 75).
- The voxel dosimetry approach is more and more applied, with potential improvement of predictive power of dosimetry. DVHs are derived, the degree of non-uniformity analyzed (17, 50, 51), and the EUBED, EUD, EQ2 parameters evaluated. Recently, the “planning target volume” concept has been proposed with D70 and V100 as parameters for response and toxicity as for EBRT (22).
- There is clinical evidence of a same biological effect (toxicity and efficacy) related to different median absorbed doses for glass and resin microspheres (110–120 Gy versus 45–50 to the single lobe) following the different number of spheres per GBq, i.e., per Gy (2, 84).
- In glass spheres, the utility of ^99m^Tc-MAA-SPECT/CT dosimetry has been confirmed in predicting both response and survival, and adapting the treatment planning especially in patients with large *T* (14, 21). An absorbed dose of 205 Gy represents the threshold for improved survival. Based on the dose distribution analysis, some patients could benefit of a boosted RE without increased G3 liver toxicity.

## Conclusion

The proposed review offers a comprehensive summary of the results and shows many successful steps reached when dosimetry and radiobiological models have been used. Methods from EBRT are ready to be inherited and/or adapted to RE applications. Some dose–effects correlations are still weak or improvable, but others have been robustly found, allowing to predict toxicity, response, and survival. Individualized dosimetric treatment planning in RE is feasible. This might definitely improve the management of primary and metastatic liver cancer.

## Conflict of Interest Statement

The editorial costs of the paper submitted will be payed by the SIR-Spheres company.
